# A Network Pharmacology Approach to Determine Active Compounds and Action Mechanisms of Ge-Gen-Qin-Lian Decoction for Treatment of Type 2 Diabetes

**DOI:** 10.1155/2014/495840

**Published:** 2014-01-16

**Authors:** Huiying Li, Linhua Zhao, Bo Zhang, Yuyu Jiang, Xu Wang, Yun Guo, Hongxing Liu, Shao Li, Xiaolin Tong

**Affiliations:** ^1^MOE Key Laboratory of Bioinformatics, Bioinformatics Division, TNLIST/Department of Automation, Tsinghua University, Beijing 100084, China; ^2^Guang'anmen Hospital, China Academy of Chinese Medical Sciences, Beijing 100053, China; ^3^Tianjin International Joint Academy of Biotechnology & Medicine, Tianjin 300457, China; ^4^Wuxi Medical School, Jiangnan University, Wuxi 214122, China

## Abstract

Traditional Chinese medicine (TCM) herbal formulae can be valuable therapeutic strategies and drug discovery resources. However, the active ingredients and action mechanisms of most TCM formulae remain unclear. Therefore, the identification of potent ingredients and their actions is a major challenge in TCM research. In this study, we used a network pharmacology approach we previously developed to help determine the potential antidiabetic ingredients from the traditional Ge-Gen-Qin-Lian decoction (GGQLD) formula. We predicted the target profiles of all available GGQLD ingredients to infer the active ingredients by clustering the target profile of ingredients with FDA-approved antidiabetic drugs. We also applied network target analysis to evaluate the links between herbal ingredients and pharmacological actions to help explain the action mechanisms of GGQLD. According to the predicted results, we confirmed that a novel antidiabetic ingredient from *Puerariae Lobatae radix* (Ge-Gen), 4-Hydroxymephenytoin, increased the insulin secretion in RIN-5F cells and improved insulin resistance in 3T3-L1 adipocytes. The network pharmacology strategy used here provided a powerful means for identifying bioactive ingredients and mechanisms of action for TCM herbal formulae, including Ge-Gen-Qin-Lian decoction.

## 1. Introduction

Type 2 diabetes (T2D), or noninsulin-dependent diabetes mellitus, is a common complex disease with an increasing prevalence worldwide. In 2012 it was estimated that more than 371 million people have diabetes and that T2D constitutes over 90% of diabetic patients [1]. Furthermore, epidemiological survey analysis suggests that the prevalence of diabetes is accelerating [2]. T2D is characterized by high blood glucose levels due to insufficient insulin secretion, insulin resistance, and impaired insulin action [3]. T2D is influenced by lifestyle factors, such as age, pregnancy, and obesity, but has a strong genetic predisposition [4]. Multiple genes are involved in genetic susceptibility, each making a small contribution to T2D risk [5, 6]. Alterations in multiple signaling pathways, for example, JAK-STAT, MAPK, VEGF, PPAR, P13K, and Wnt were implicated in the pathogenesis of the disease [7–9]. Treatments aimed at controlling high-level blood glucose, as well as therapies that prevent diabetic complications, have all shown specific therapeutic activity in T2D patients, such as metformin, alpha-glucosidase inhibitors, sulfonylureas, thiazolidinediones (TZDs), and insulin injections [10]. However, these treatments have shown limited efficacy and are associated with various side effects such as flatulence and diarrhea [11]. Therefore, as a complicated disease, T2D may require complex therapeutic approaches such as traditional Chinese medicine (TCM) [12].

In TCM, T2D is treated as “Xiaoke” and the related herbal formulae have been used over thousands of years. The therapeutic effects of Chinese medicines used for the treatment of T2D have been documented based on clinical trials or the use of animal T2D models [13, 14]. One such herbal formula is the Ge-Gen-Qin-Lian decoction (GGQLD), an ancient and effective treatment for “dampness-heat” ZHENG causing diarrhea and dysentery, which originated from “*Shanghan Lun*” compiled by ZhongJing Zhang. GGQLD consists of four herbs: *Puerariae Lobatae radix* (Ge-Gen) as the principle herb, *Scutellariae radix* (Huang-Qin), *Coptidis rhizoma* (Huang-Lian), and *Glycyrrhizae Radix et Rhizoma Praeparata cum Melle* (Gan-Cao) used as adjuvant herbs to assist the effects of Ge-Gen. It has been reported that puerarin from Ge-Gen reduced blood sugar in diabetic mice, and improved insulin resistance and hyperlipidemia in rats [15–17]. Baicalin from Huang-Qin had antihyperglycemic effects on diabetic rats [18]. Berberine from Huang-Lian lowered blood glucose significantly by increasing insulin receptor expression [19]. Furthermore, amorfrutins from Gan-Cao have potent antidiabetic activity [20]. Intriguingly, recent studies also showed that GGQLD had good clinical effects on T2D and the anti-diabetic activities of GGQLD *in vivo* and *in vitro* were investigated [21, 22]. Although research has indicated that GGQLD, composed of multiple biologically active compounds, helps in T2D treatment, the mechanism of this formula remains unknown due to its complex nature, as well as a lack in appropriate methods.

As complex mixtures of herbs, TCM formulae consist of many small molecular compounds, which may simultaneously, transiently (short residue time), or weakly (low affinity) bind with multiple target proteins [23, 24]. The systematic therapeutic strategy of a formula is realized through collectively targeting the disease-specific molecular network. Its increased efficacy and decreased toxicity may arise as a result of complex synergistic or antagonistic interactions among different formula components. Such features of TCM herbal formulae meet the requirements of complex disease (e.g., T2D) treatment in a systematic manner. Interestingly, the holistic philosophy of TCM is consistent with the key idea of emerging network pharmacology [25, 26]. Recently, TCM network pharmacology has been proposed by Li et al. [25, 27–30], which integrates TCM theory with interaction networks and uses a “network target” as a mathematical and computable representation of various connections between herbal formulae and diseases [31, 32]. The combinatorial rules and holistic regulation effects of herbal formulae can be conveyed using the network properties arising directly from network topology and dynamics, such as the network parameters including connectivity, centrality, modularity, and propagation [33–37]. Therefore, TCM network pharmacology can be used to understand the scientific basis of TCM herbal formulae at the molecular level and from a system perspective.

Our previous studies have shown that the “network target” as a key concept of TCM network pharmacology can help to decipher the molecular mechanisms of the therapeutic effects of TCM herbal formulae and to determine their active ingredients or combinations [25, 31, 32, 38]. Here, to better understand the molecular basis of the therapeutic effects of GGQLD on T2D, we computationally recognized the active ingredients and mechanisms in GGQLD using integrative analysis based on our TCM network pharmacology platform and experimentally validated the antidiabetic activity of the candidate ingredients. Network target analysis showed that GGQLD can regulate key biological processes in T2D development, such as glucose homeostasis and response to insulin stimulus. Moreover, we revealed that 4-Hydroxymephenytoin, a core component of Ge-Gen, was involved in the antidiabetic ingredients of GGQLD, which can stimulate endogenous insulin secretion and ameliorate insulin resistance in 3T3-L1-based insulin resistance models. These results provide new insight into the molecular mechanisms of the antidiabetic activity of GGQLD and accelerate drug discovery on the basis of GGQLD.

## 2. Methods and Materials

### 2.1. Computational Prediction of Antidiabetic Ingredients from GGQLD Using Network Target Analysis

#### 2.1.1. Data Collection

We collected the TCM herbal ingredients imported from the Herb BioMap database (China Copyright of Computer Software, 2011SR076502), which contains information on 621 herbs and 10,805 distinct chemical ingredients. To identify the active ingredients in GGQLD, a total of 287 available chemical ingredients were collected, with 42 found in Ge-Gen, 57 found in Huang-Qin, 22 found in Huang-Lian, and 166 found in Gan-Cao. The chemical information on GGQLD ingredients (structure, canonical name, and CID number) employed for computational analysis was downloaded from the PubChem Compound database (http://pubchem.ncbi.nlm.nih.gov/) [39]. A data set of 80 T2D-related genes and the true targets of 19 FDA-approved antidiabetic drugs were retrieved from the OMIM Morbid Map and DrugBank databases, respectively [40, 41].

#### 2.1.2. Network-Based Prediction of Herbal Ingredient Target Profiles

*In silico* prediction of comprehensive target profiles of TCM ingredients is the first step in TCM network pharmacology. Compared with virtual screening based on docking analysis, the network-based computational approach for drug target identification is not restricted to the target protein structures. In this study, a network-based regression model (drugCIPHER) for target profile prediction was carried out. The drugCIPHER method scored the likelihood of drug-target interactions by integrating structural similarities of drugs and protein-protein interactions in a heterogeneous network that correlated chemical and genomic spaces [42]. Briefly, drugCIPHER was performed to predict the target profiles of each GGQLD ingredient. The drugCIPHER score represented the likelihood of an ingredient-target interaction, which was obtained from the correlation between the query ingredient's structural similarity vector in chemical space and the target protein's closeness vector in genomic space. Finally, the top 100 proteins were selected as target profiles for each ingredient since the top 100 targets reach the high prediction accuracy (77.3%) in general and can be a representation of the whole target profile [42].

#### 2.1.3. Cluster Analysis of Target Profiles for Computational Identification of Antidiabetic Ingredients

Two-dimensional hierarchical clustering of target profiles was used to determine possible antidiabetic ingredients by comparing the GGQLD ingredients and antidiabetic drug profiles [43]. For the purpose of comparison and cluster analysis, 19 FDA-approved antidiabetic drugs were compiled (Table 1) and their target profiles were generated using drugCIPHER. Clustering was executed using MATLAB (Mathworks Matlab R2013a) and standard hierarchical clustering of a matrix dependence on drugs or ingredient target profiles. The clustering coefficient between GGQLD ingredients and antidiabetic drug profiles was estimated and the cutoff value was set >0.5. The similarity networks of herbal ingredients and drugs (clustering coefficient of target profiles >0.5) were created using network visualization software CytoScape 2.8.

#### 2.1.4. Network Pharmacology for Predicting Synergistic Herbal Ingredient Combinations

Prediction of synergistic herbal ingredient combinations was performed as described previously [32]. We adapted a synergy score that predicted how strong disease molecular networks were perturbed with herbal ingredient combinations. In this study, berberine in Huang-Lian was used to search possible synergistic combinations with the 286 GGQLD ingredients. Different features of the disease molecular network were combined to quantify the synergy, such as node importance and shortest path distance between berberine and other ingredient target profiles. The calculation details have been described previously [32]. Statistical significance of the synergistic herbal ingredient combinations was estimated using *P* value.

#### 2.1.5. Network Target Analysis of Mechanisms of Action of GGQLD

Identification and characterization of the action mechanism of GGQLD were performed using network target analysis as depicted previously [38]. Briefly, a gene ontology (GO) enrichment analysis tool from the DAVID database was used to identify statistically significant enriched GO biological process (BP) terms of assembled T2D-related genes (*P* value < 0.05 after Benjamini's correction), which were selected as common biological functions and pathways for T2D [44]. Secondly, the target profile of each antidiabetic GGQLD ingredient inferred by cluster analysis and the gene set of enriched GO BP terms of T2D-related genes were mapped simultaneously onto the protein-protein interaction (PPI) network assembled PPI data from public databases [38]. Thirdly, the possible biological functions of each antidiabetic ingredient were predicted by the network-based algorithm of drug function identification developed previously, which integrated important network parameters such as node importance and shortest path distance between the herbal ingredients' target gene set and T2D-related gene set [45]. Fourthly, we constructed a large GGQLD herbal ingredient-function network with GO BP terms and herbal ingredients as nodes and the links extracted from the *P* value calculated above. Lastly, a more biologically meaningful subnetwork representing the action mechanisms of GGQLD was produced, which was visualized by CytoScape software.

### 2.2. Experimental Validation for Computational Prediction Results Using Insulin Secretion Assay and Insulin Resistance Model

#### 2.2.1. Drugs and Reagents

Two FDA-approved antidiabetic drugs (nateglinide and pioglitazone), a known antidiabetic herbal ingredient (berberine), the newly discovered GGQLD herbal antidiabetic (4-Hydroxymephenytoin), and palmitic acid (PA, P0500) were obtained from Sigma Chemical Co. (St. Louis, Missouri, USA). The RPMI 1640 medium, high-glucose DMEM medium, 3-isobutyl-1-methylxanthine, trypsin, and fetal bovine serum for cell culture were purchased from GIBCO (Grand Island, New York, USA). The MTT detection kit, insulin ELISA kit, and glucose detection kit were obtained from R&D (Minneapolis, USA).

#### 2.2.2. Cell Cultures and Treatment

RIN-5F cells derived from rat insulinoma and 3T3-L1 preadipocytes derived from 3T3 mouse embryo fibroblast were purchased from the American Type Culture Collection (Manassas, VA; ATCC no.: CRL-2058 and CL-173, resp.). The RIN-5F cells were cultured in RPMI 1640 medium containing 10% FBS, 100 U/mL penicillin, and 100 *μ*g/mL streptomycin under an atmosphere of 5% CO_2_/95% humidified air at 37°C. The medium was renewed every 3 days. The cells were used at passages 20–25. The 3T3-L1 preadipocytes were grown and differentiated into adipocytes as described previously [46]. Briefly, preadipocytes were differentiated in high-glucose DMEM, 10% FBS with dexamethasone (0.25 *μ*M), insulin (10 *μ*g/mL), and 3-isobutyl-1-methylxanthine (0.5 mM) for 48 h and then treated with insulin (1 *μ*g/mL) for an additional 48 h. Adipocytes were maintained in and refed every 2 days with high-glucose DMEM and 10% FBS until being used for experiments 8–12 days after the addition of differentiation factors, when between 90 and 95% of cells exhibited an adipocyte phenotype. For antidiabetic drugs or herbal ingredient treatment of cells, RIN-5F and 3T3-L1 adipocytes were cultured for the indicated time in the corresponding medium containing BSA (2%) and glucose (5.4 mM) in the absence of nateglinide, pioglitazone, berberine, and 4-Hydroxymephenytoin. After that, other assays were performed.

#### 2.2.3. MTT Assay

A total of 1 × 10^4^ cells were plated in 96-well flat-bottom plates in 100 *μ*L of medium. The next day, cells were exposed to the antidiabetic drugs and potential herbal ingredients at different concentrations. After one day from the last drug addition, 20 *μ*L of 5 mg/mL MTT solution in PBS was added to each well for 4 h. The medium was removed, and 200 *μ*L DMSO was added to each well to dissolve the formazan crystals. Absorption at 570 nm was determined using a Bio Rad microplate reader (Model 3550 microplate reader, Bio-Rad Laboratories, Richmond, CA). Triplicate wells were assayed for each condition, and standard deviations were determined. The concentrations of each agent with survival rates >90% were selected for use in the following assays.

#### 2.2.4. Insulin Secretion

The RIN-5F cells were seeded in 12-multiwell plates at a density of 10^7^ cells/mL. After 24 h, the medium was discarded, and the cells were washed twice for 30 min at 37°C with PBS. The cells were incubated in the presence or absence of increasing concentrations of glucose for different periods of time. To study herbal ingredient-induced insulin secretion, RIN-5F cells were incubated for the last indicated time at 37°C in the medium with different glucose content in the presence or absence of different concentrations of nateglinide, berberine, and 4-Hydroxymephenytoin. Aliquots of the supernatant were collected and stored at −20°C for subsequent insulin amount determination by ELISA.

#### 2.2.5. Oil Red O Staining

Oil red O was used to stain lipids in the 3T3-L1 adipocytes. Cells were washed three times with PBS and then once in 60% isopropanol. Oil red O was added and the cells were incubated for 20 min at room temperature. Cells were washed three times in PBS and then once in 60% isopropanol again. Slides were rinsed and counterstained with haematoxylin. Mounting solution and coverslips were added.

#### 2.2.6. Insulin-Resistance Model and Glucose Consumption Assay

The 3T3-L1 adipocytes were cultured for 12 h in serum-free DMEM with 0.2% BSA. The cells were then cultured in DMEM containing 1% BSA for 24 h (normal group); or in DMEM containing 0.5 mM PA and 2% BSA (model group); or in DMEM containing 0.5 mM PA, 0.1, 1, and 10 *μ*M pioglitazone, berberine, and 4-Hydroxymephenytoin, and 2% BSA (treatment group) for 24 h [47]. The 3T3-L1 preadipocytes were differentiated to adipocytes in a 12-well plate. After serum starvation in 0.2% BSA DMEM overnight, the cells were incubated with DMEM containing 0.5 mM PA and various concentrations of pioglitazone, berberine, and 4-Hydroxymephenytoin for 24 h. The medium was removed and its glucose concentrations were determined by the glucose oxidase method. The amount of glucose consumption was calculated by subtracting the remaining glucose in the plate from the glucose concentrations of blank wells. Three replicate wells were established. Additionally, an MTT assay was employed to determine cell number and viability.

#### 2.2.7. Statistical Analysis

Data were expressed as means ± standard deviation (SD). Multigroup comparisons were carried out by analysis of variance (ANOVA) with SPSS 16.0. Values of *P* < 0.05 were considered statistically significant.

## 3. Results and Discussion

### 3.1. Computational Prediction Based on Network Pharmacology for GGQLD

Cells employ complex signaling networks to drive biological processes. Genetic or epigenetic alterations in signaling pathways and networks might result in imbalanced signaling of islet cells, which then leads to T2D phenotypes. The successful application of GGQLD in T2D therapy not only demonstrated the feasibility of TCM herbal formulae but also showed that increasing the complexity of proposed therapies by targeting different pathological processes in disease development should more efficiently treat complex diseases. Interestingly, the network-based therapeutic strategies are evidence based presumably in agreement with the properties of TCM herbal formulae [48]. Previously, we showed that integrative TCM network pharmacology and its application provided insight into the combinatorial role of an antirheumatoid arthritis herbal formula and identified the active ingredients [38]. To gain further insight into the underlying mechanism of GGQLD, we applied a network-based approach to predict the target profiles of each herbal GGQLD ingredient and 19 FDA-approved antidiabetic drugs (Table 1). Here, the target profiles of herbal compounds and 19 FDA-approved antidiabetic drugs will be compared and clustered to predict the actions of herbal compounds. Note that although the FDA-approved drugs have their known targets, we need to predict the target profile of these FDA drugs by drugCIPHER, making the target and activity information more complete and more comprehensive for the FDA drugs. The reliability and precision of the target profiles predicted by drugCIPHER have been evaluated for FDA-approved small molecular drugs [42]. The genes involved in target profiles were defined by all genes within the assembled PPI network. We also restricted our analysis to the top 100 genes present in each target profile.

#### 3.1.1. Identifying Antidiabetic Ingredients from GGQLD Using Cluster Analysis

**Figure 1:**
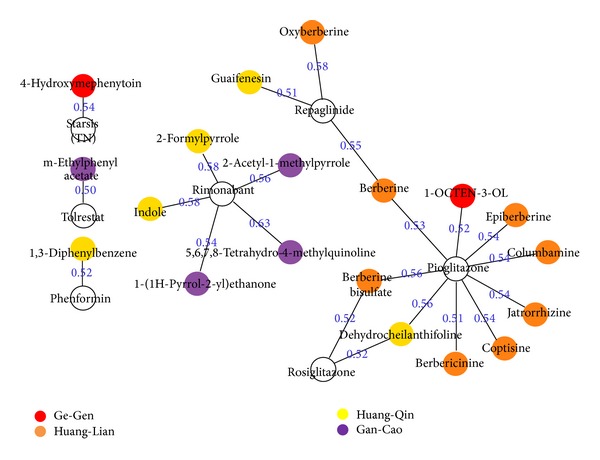
The herbal ingredient-drug networks based on the target profile cluster analysis. Each node represents a herbal ingredient or drug. The color of herbal ingredient represents its source herbs. Two nodes between herbal ingredients and drugs are linked by an edge if their similarity score is above the predefined threshold >0.5.

To computationally determine antidiabetic herbal ingredients and understand the molecular basis of GGQLD, we hypothesized that the potential antidiabetic herbal ingredients and FDA-approved antidiabetic drugs were likely to share similar target profile patterns or similar biological functions and pharmacological action. In line with prior research [38], we measured the similarity between the target profiles of herbal ingredients and the selected drugs using the hierarchical clustering algorithm, which revealed that certain herbal ingredients were associated with antidiabetic drugs with diverse action mechanisms. After cluster analysis using target profiles, the herbal ingredients with coefficients of more than 0.5 were analyzed in greater detail and a similarity network with weighted edges based on differences in the target profiles of herbal ingredients and drugs was generated (Figure 1). This network represented similar relationships or common mechanisms between herbal ingredients and drugs.  Table 2 shows that 19 herbal ingredients in this network were identified as potential antidiabetic ingredients in GGQLD, of which two, nine, four, and four ingredients were from Ge-Gen, Huang-Lian, Huang-Qin, and Gan-Cao, respectively. To demonstrate whether these ingredients had antidiabetic properties, a literature search was performed using SciFinder [49] and PubMed. This search identified 13 of the 19 herbal ingredients as known antidiabetic compounds supported by at least one independent item of evidence [50–63]. These ingredients were linked to diverse antidiabetic activities in previous studies and were computationally confirmed here. For example, 1-OCTEN-3-OL from Ge-Gen and 2-Acetyl-1-methylpyrrole from Gan-Cao were found to be antioxidants involved in the improvement of diabetes [50, 63, 64]; guaifenesin from Huang-Qin promoted neurite outgrowth and protected diabetic mice from neuropathy [61]. In particular, several core ingredients from Huang-Lian, berberine bisulfate, columbamine, coptisine, epiberberine, jatrorrhizine, oxyberberine, dehydrocheilanthifoline, and berberine were reported to have hypoglycemic and antidiabetic actions through the regulation of glucose metabolic effects and reduction of oxidative stress injury [51–59]. These results demonstrated the reliability of our approach and provided possible explanations for the molecular basis and mechanisms of action of GGQLD [21, 22]. For the remaining six ingredients not reported in the literature, 4-Hydroxymephenytoin was commercially available and was tested in *in vitro* antidiabetic assay below.

#### 3.1.2. Determining Combinatorial Rules of GGQLD Using Network Pharmacology

From the computational screening of the pooled GGQLD herbal ingredients, a known antidiabetic ingredient (berberine in Huang-Lian) was selected as a core component to combine with other ingredients, resulting in 287 unique ingredient pairs. We used significant synergy scores to identify potent ingredient pairs.  Table 3 shows the potent berberine-ingredient pairs with significant *P* values (*P* < 0.05). Berberine treats T2D by lowering blood glucose and improving insulin-resistant states. The significant top ranked herbal ingredient pairs suggest that berberine combined with oxyberberine in Huang-Lian and guaifenesin in Huang-Qin may produce synergistic antidiabetic actions. This result was indirectly validated by the synergistic combinations of FDA-approved antidiabetic drugs with similar target profiles. For example, the combination of repaglinide and pioglitazone has acceptable safety with greater reductions in glycemic parameters than treatment using either agent alone [65]. In addition, the combinational effects of berberine with other ingredients in GGQLD were also predicted, which needs further experimental verification. We suspect that the synergistic mechanisms of berberine with 5,6,7,8-Tetrahydro-4-methylquinoline/1-(1H-Pyrrol-2-yl)ethanone in Gan-Cao or indole in Huang-Qin might be due to their different antidiabetic mechanisms, as the similarity network found the difference of target profiles.

#### 3.1.3. Understanding the Action Mechanisms of GGQLD Using Network Target Analysis

**Figure 2:**
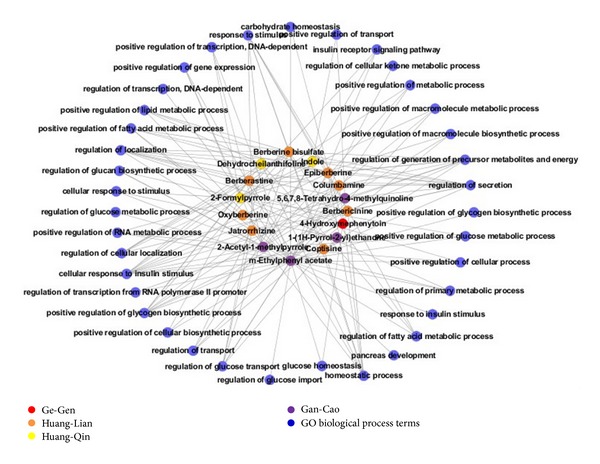
Relationships between antidiabetic ingredients and their perturbed GO Biological Processes. Blue nodes represent T2D-related GO Biological Processes; other color nodes represent ingredients from different herbs. This network can be regarded as a perturbed network targeted by various ingredients from GGQLD.

Comodule analysis by mapping disease genes and drug target profiles into the integrative PPI network suggests an underlying link between disease and drugs if the two gene sets coexist within the same module or the distance between them is very close in the network. The network-based approach could therefore provide predictable power for the pharmacological actions of herbal ingredients. Consequently, to identify which T2D-related biological processes were regulated by the predicted herbal ingredients from GGQLD, network target analysis was performed with the T2D-related genes enriched GO terms and target profiles of herbal ingredients. Because the network distribution of T2D genes was considered a specific network target, the relationship between T2D pathological processes and herbal ingredients was evaluated using node importance and shortest path distance, as previously described [45]. The network target analysis enabled a comprehensive understanding of the action mechanisms of GGQLD so that the resulting network could be used to explain the combinatorial activities of herbal ingredients in GGQLD.  Figure 2 shows that the key biological processes involved in T2D (glucose homeostasis, regulation of glucose import, regulation of glucose transport, regulation of glucose metabolic process, and response to insulin stimulus) were regulated by different ingredients from GGQLD. This suggests that GGQLD can treat T2D by regulating the complex network related to multiple pathological processes of T2D.

Furthermore, our previous studies found that Cold ZHENG and Hot ZHENG in the classic theory of TCM were characterized by imbalance in the metabolic and immune networks, considering from a biological or molecular correlate between the ZHENG and diseases [27, 66, 67]. There are two therapeutic strategies, one direct and one indirect, for attempting to recover the Yin and Yang balance in the human body. For example, “clearing heat” is used in the direct treatment of Hot ZHENG, while the therapeutic principle of “nourishing Yin and clearing heat” is used in the indirect treatment of Hot ZHENG. GGQLD with cold-dominated herbs is now routinely used for treating “damp-heat syndrome” in TCM clinics. As shown in Figure 2, GGQLD mainly affected the positive regulation of many types of metabolic processes, suggesting that GGQLD can efficiently treat T2D by improving the imbalanced state of metabolic processes in T2D patients. These results showed that the therapeutic strategy of GGQLD is indirect and also implied that the “damp-heat syndrome” treated by GGQLD may be one of the phenotypes caused by “Yin deficiency,” such as the reduced secretion of insulin.

### 3.2. Experimental Validation of the Antidiabetic Effect of 4-Hydroxymephenytoin

**Figure 3:**
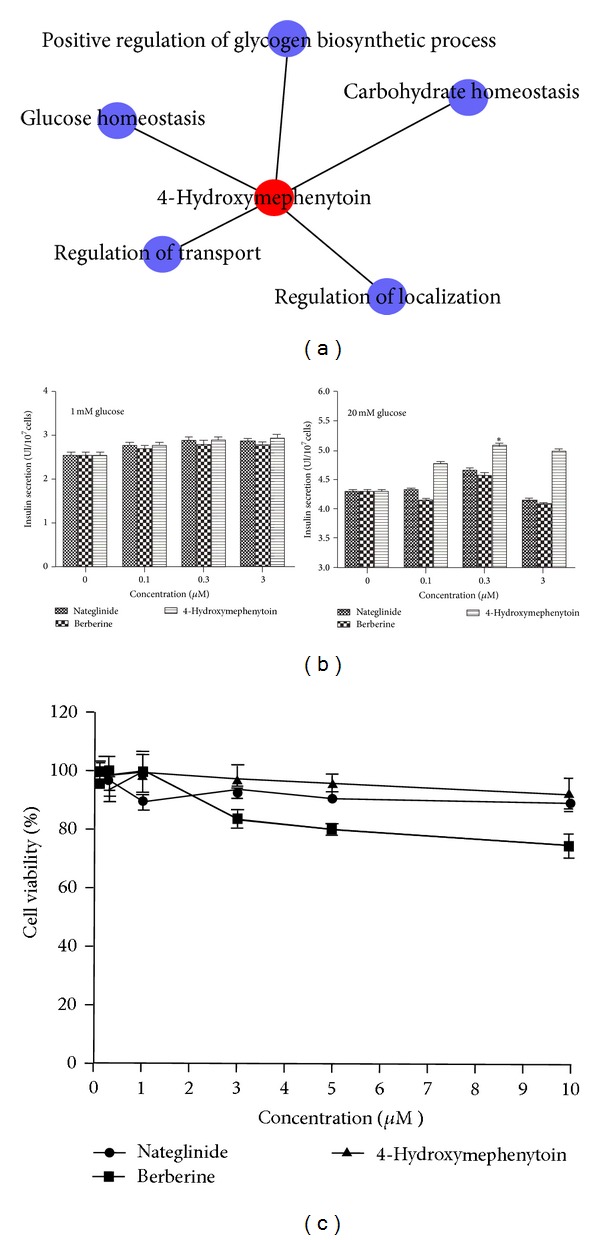
4-Hydroxymephenytoin induced insulin secretion in RIN-5F cells at a nontoxic concentration. (a) Subnetwork targeted by 4-Hydroxymephenytoin. (b) Effects of nateglinide, berberine, and 4-Hydroxymephenytoin on glucose-stimulated insulin secretion from RIN-5F islet cells. Cells were incubated with various concentrations of nateglinide, berberine, and 4-Hydroxymephenytoin for 2 h to induce insulin secretion. Glucose with different concentrations was used as the control for basal and hyperglycemic conditions. Values are means ± SD of five replicate experiments in each group. *P < 0.05 and **P < 0.01 are compared with the control group of 20 mM glucose. (c) Effects of nateglinide, berberine, and 4-Hydroxymephenytoin on cytotoxicity in RIN-5F islet cells. Cells were incubated with various concentrations of nateglinide, berberine, and 4-Hydroxymephenytoin, respectively for 24 h in the presence of 20 mM glucose. Cell viability was measured using MTT assay. Values are means ± SD of seven replicate experiments in each group.

Patients who develop T2D have a complex phenotype with disordered insulin secretion, increased hepatic glucose production, and resistance to the action of insulin, which all contribute to the development of overt hyperglycemia [68]. The therapeutic action mechanisms of antidiabetic agents on T2D might be related to their effect of ameliorating glucose metabolism disorders, improving insulin resistance, and increasing tissue sensitivity to insulin. In this study, we investigated the activities of 4-Hydroxymephenytoin on insulin secretion and resistance *in vitro* to validate its antidiabetic potential, as shown in Figure 3(a).

#### 3.2.1. Effect of 4-Hydroxymephenytoin on Insulin Secretion

To assess the stimulatory effect of 4-Hydroxymephenytoin on insulin secretion *in vitro*, we used a rat insulin enzyme-immunoassay to determine activity based on the insulin levels released from RIN-5F cells under both basal and hyperglycemic conditions, which contained 1 and 20 mM glucose, respectively. As shown in Figure 3(b), insulin secretion was augmented when the glucose concentration increased from 1 to 20 mM. Although nateglinide, berberine, and 4-Hydroxymephenytoin in the presence of 1 mM glucose did not significantly induce insulin secretion from the cells, exposure to 4-Hydroxymephenytoin (0.1, 0.3, and 3 *μ*M) in the presence of 20 mM glucose stimulated a significant increase in insulin secretion in a concentration-dependent manner (*P* < 0.01). Interestingly, RIN-5F cells responded better to 4-Hydroxymephenytoin than to nateglinide and berberine in insulin secretion. Maximum activity was observed with 0.3 *μ*M of 4-Hydroxymephenytoin. At 20 mM glucose, 4-Hydroxymephenytoin induced a 1.9-fold increase in insulin secretion compared to that without 4-Hydroxymephenytoin, whereas 4-Hydroxymephenytoin in 1 mM glucose showed lower activity. Toxicity profiles of nateglinide, berberine, and 4-Hydroxymephenytoin in the RIN-5F cells were determined at 0.1–10 *μ*M of nateglinide, berberine and 4-Hydroxymephenytoin by cell proliferation assay (Figure 3(c)). The incubation period for the induction of insulin secretion was insufficient to detect cytotoxicity. Therefore, the cultures were incubated for 24 h. 4-Hydroxymephenytoin did not show apparent cytotoxicity up to 1 *μ*M, indicating that the activities of 4-Hydroxymephenytoin on insulin secretion were not due to their toxicity. Moreover, toxic concentrations of 4-Hydroxymephenytoin were higher than these of nateglinide and berberine.

#### 3.2.2. Improvement of 4-Hydroxymephenytoin on Insulin Resistance and Glucose Consumption

**Figure 4:**
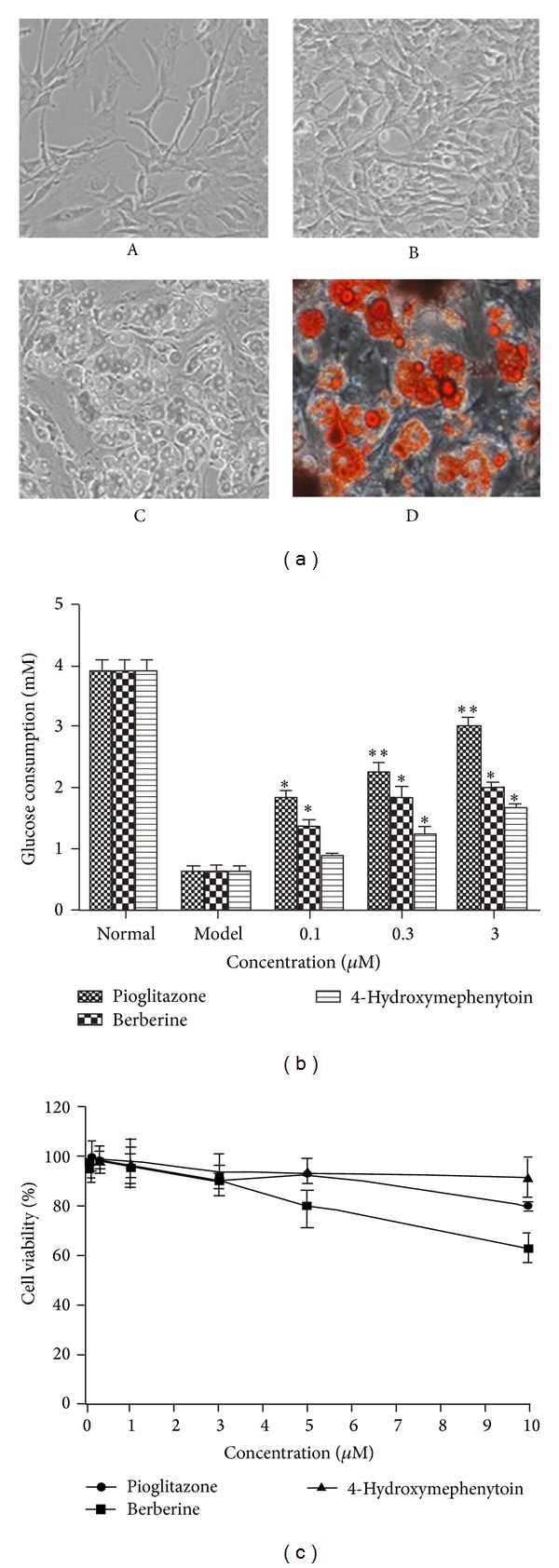
4-Hydroxymephenytoin induced insulin-induced glucose consumption in 3T3-L1 adipocytes. (a) General view of differentiation of 3T3-L1 preadipocytes into adipocytes as seen in inverted phase contrast microscope (A–D). Large lipid droplets are the main characteristics of the cytoplasm of the cells (C). Intracellular lipid content was measured by oil red O staining. (b) Effects of pioglitazone, berberine, and 4-Hydroxymephenytoin on glucose consumption in 3T3-L1 adipocytes. Differentiated 3T3-L1 adipocytes in 96-well plates were preincubated with DMEM containing 0.2% BSA for 12 h and then incubated with various concentrations (0.1, 0.3, and 3 *μ*M) of pioglitazone, berberine, and 4-Hydroxymephenytoin for 24 h. Glucose consumption amount was obtained from the difference in glucose concentrations between initial and final states for the indicated time from the culture medium. Values are means ± SD of five replicate experiments in each group. *P < 0.05 and **P < 0.01 are compared with model group. (c) Effects of pioglitazone, berberine, and 4-Hydroxymephenytoin on cytotoxicity in 3T3-L1 adipocytes. Cells were incubated with various concentrations of pioglitazone, berberine, and 4-Hydroxymephenytoin for 24 h. Cell viability was measured using MTT assay. Values are means ± SD of seven replicate experiments in each group.

We investigated the effects of 4-Hydroxymephenytoin on improving insulin resistance induced by PA in 3T3-L1 adipocytes. Differentiated 3T3-L1 adipocytes from preadipocytes were used as a cellular model to evaluate the activity of 4-Hydroxymephenytoin for the improvement of insulin resistance. Adipocyte differentiation was induced in the 3T3-L1 preadipocytes using 3-isobutyl-1-methylxanthine and dexamethasone, and lipid droplets were detected with oil red O staining. Because increased adiposity in the 3T3-L1 adipocytes may be due to increased adipocyte differentiation, adipogenesis is shown in Figure 4(a). The 3T3-L1 adipocytes were treated with PA (0.5 mM, 24 h) to induce insulin resistance. Insulin-induced glucose consumption was measured to determine insulin sensitivity. The results showed that insulin-induced glucose consumption was ~8 times higher than that in the normal group but was inhibited by as much as 87.5% after incubation with PA for 24 h, which was consistent with previous free-fatty acid-induced insulin resistance studies [69]. However, intervention with 0.1, 0.3, and 3 *μ*M of pioglitazone, berberine and 4-Hydroxymephenytoin reversed the condition somewhat. Insulin-induced glucose consumption was increased by 43.8%, 100%, and 162.5% after intervention with 0.1, 0.3 and 3 *μ*M of 4-Hydroxymephenytoin, respectively for 2 h and were both dose- and time-dependent. In addition, the insulin-induced glucose consumption increased by 368.8% and 212.5%, respectively, in the 3 *μ*M pioglitazone and berberine groups (Figure 4(b)). As shown in Figure 4(c), nontoxic concentrations of each agent were selected in this study. Together, these results suggest that 4-Hydroxymephenytoin can improve insulin resistance and increase glucose consumption in the 3T3-L1 adipocytes, albeit less efficiently than pioglitazone and berberine at 0.3 *μ*M.

## 4. Conclusions

A practical application of the network-based approach was illustrated in GGQLD and the results demonstrate that this approach is an effective strategy for TCM modern research. Increased coverage, quality, and variety of herbal ingredients data involved in each herb will, in turn, enable further opportunities for molecular basis of TCM herbal formulae. Integrating chemical, target protein binding, gene and protein expression, pharmacokinetic and pharmacodynamic or diagnostic and clinical information hold further promise for determining relationships between diseases and TCM herbal formula at multiple levels. At present, the success of network-based active ingredient identification and mechanism prediction supports the notion that TCM herbal formulae target a disease-specific network [26] and that the key to understanding the mechanisms of action and combinatorial rules of TCM herbal formulae is encoded in the molecular network. The antidiabetic activities of the GGQLD herbal ingredients were identified in this work through network pharmacology methods and can serve as potential antidiabetic ingredients for future experimental validation, and 4-Hydroxymephenytoin from Ge-Gen is such a representation validated in this study.

## Figures and Tables

**Table 1:** Nineteen FDA-approved antidiabetic drugs compiled from the Drugbank database.

Category	Name	CID
Sulfonylureas drug	Tolbutamide	5505
	Glyburide	3488
	Glipizide	3478
	Gliquidone	91610
	Glimepiride	3476
	Gliclazide	3475

Biguanide drug	Metformin	4091
	Phenformin	8249
	Acarbose	441184
	Voglibose	444020
	Miglitol	441314

Euglycemic agent	Pioglitazone	4829
	Rosiglitazone	77999

Euglycemic agent (Nonsulfonylurea)	Repaglinide	65981
	Starsis (TN)	443871

Aldose reductase inhibitor	Tolrestat	53359
	Alrestatin	2120

Other oral antidiabetic drugs	Sitagliptin	4369359
	Rimonabant	104850

**Table 2:** Potential antidiabetic ingredients in Ge-Gen-Qin-Lian formula by network target analysis.

Ingredients	Herbs	CID	Literature evidence
4-Hydroxymephenytoin	Ge-Gen	119507	/
1-OCTEN-3-OL	Ge-Gen	18827	[50]
Berbericinine	Huang-Lian	19009	/
Berberine bisulfate	Huang-Lian	12457	[51]
Columbamine	Huang-Lian	72310	[52]
Coptisine	Huang-Lian	72322	[53]
Epiberberine	Huang-Lian	160876	[54, 55]
Jatrorrhizine	Huang-Lian	72323	[55, 56]
Oxyberberine	Huang-Lian	11066	[57]
Dehydrocheilanthifoline	Huang-Lian	3084708	[58]
Berberine	Huang-Lian	2353	[59]
Indole	Huang-Qin	798	[60]
1,3-Diphenylbenzene	Huang-Qin	7076	/
2-Formylpyrrole	Huang-Qin	13854	/
Guaifenesin	Huang-Qin	3516	[61]
1-(1H-Pyrrol-2-yl)ethanone	Gan-Cao	14079	[62]
2-Acetyl-1-methylpyrrole	Gan-Cao	61240	[63]
m-Ethylphenyl acetate	Gan-Cao	76462	/
5,6,7,8-Tetrahydro-4-methylquinoline	Gan-Cao	185667	/

/: no evidence.

**Table 3:** Herbal ingredients in GGQLD with potential synergistic antidiabetic effects on berberine.

Herb name	Chemical name	Synergy score	*P* value
Gan-Cao	5,6,7,8-Tetrahydro-4-methylquinoline	0.532	0.001
Huang-Qin	Indole	0.463	0.001
Ge-Gen	SA3	0.452	0.001
Huang-Lian	Oxyberberine	0.372	0.006
Huang-Qin	Guaifenesin	0.453	0.011
Gan-Cao	1-(1H-Pyrrol-2-yl)ethanone	0.359	0.042
